# Efficacy and Safety of the Combination of Transarterial Chemoembolization with Camrelizumab plus Apatinib for Advanced Hepatocellular Carcinoma: A Retrospective Study of 38 Patients from a Single Center

**DOI:** 10.1155/2022/7982118

**Published:** 2022-05-09

**Authors:** Jin-Xing Zhang, Yu-Xing Chen, Chun-Gao Zhou, Jin Liu, Sheng Liu, Hai-Bin Shi, Qing-Quan Zu

**Affiliations:** ^1^Department of Interventional Radiology, The First Affiliated Hospital with Nanjing Medical University, Nanjing 210029, China; ^2^Department of Clinical Medicine Research Institution, The First Affiliated Hospital with Nanjing Medical University, Nanjing 210029, China

## Abstract

**Objective:**

To evaluate the effectiveness and safety of transarterial chemoembolization (TACE) combined with immune checkpoint inhibition (camrelizumab) plus an antiangiogenic agent (apatinib) for advanced hepatocellular carcinoma (HCC).

**Methods:**

Between March 2019 and April 2021, the clinical data of 38 patients diagnosed with advanced HCC who initially received TACE combined with camrelizumab plus apatinib were reviewed retrospectively. The objective response rate (ORR) and disease control rate (DCR) according to modified response evaluation criteria in solid tumors, progression-free survival (PFS), overall survival (OS), and adverse events (AEs) were evaluated.

**Results:**

At 2-3 months after initial therapy, the ORR and DCR was 50.0% (19/38) and 76.3% (29/38), respectively. The median PFS and OS were 7.3 months (range: 1.0–22.6 months) and 13.5 months (range: 2.3–24.3 months), respectively. Treatment-related AEs (grades 3-4) were observed in 25 patients (67.8%). No treatment-related deaths occurred.

**Conclusion:**

The combination of TACE with camrelizumab plus apatinib for the treatment of patients with advanced HCC showed promising efficacy and a manageable safety profile.

## 1. Introduction

Patients with hepatocellular carcinoma (HCC) are usually diagnosed at an advanced stage and have a large tumor burden, vascular invasion, and/or extrahepatic metastasis [1, 2]. Advanced HCC has a dismal prognosis, with a limited natural survival of 3-4 months [3]. Thus, effective management and durable remission of these advanced lesions is challenging.

The mainstay of treatment for advanced HCC is systemic therapy, with the tyrosine kinase inhibitor (TKI) sorafenib as the first-line treatment for decades [2, 4]. Other TKIs, such as lenvatinib, antivascular endothelial growth factor (VEGF), and immune checkpoint inhibition (ICI), have shown efficacy in treating advanced HCC [4–6]. Recently, combination therapy with TKIs/anti-VEGF and ICIs improved the objective response rate (ORR) to 33.2%–34.3% as a first-line treatment for advanced HCC in phase 2/3 studies [5, 7].

In Asia, locoregional therapy-transarterial chemoembolization (TACE) is one of the recommended treatment modalities for advanced HCC with well-preserved liver function [8]. TACE can target multinodular lesions and decrease the tumor burden after at one session. In addition, sequential administration of TKI/anti-VEGF and ICI after TACE could decrease neovascularization and prolong the immune activation against HCC, providing a synergistic effect and intensifying the antitumor response [9–11]. We hypothesized that TACE with apatinib plus camrelizumab could achieve better local and/or distant tumor control for patients with advanced HCC.

Here, this retrospective study aimed to evaluate the efficacy and safety of the combination of TACE with camrelizumab plus apatinib for advanced HCC patients.

## 2. Materials and Methods

### 2.1. Study Design

This was a single-center, retrospective study performed at the First Affiliated Hospital of Nanjing Medical University. This retrospective study was approved by our institutional ethics review board, and informed consent was waived. All procedures performed in studies involving human participants were in accordance with the ethical standards of the institutional and/or national research committee and with the 1964 Helsinki Declaration and its later amendments or comparable ethical standards.

### 2.2. Population

Between March 2019 and April 2021, patients with advanced HCC who were initially treated with combination of TACE and ICI (camrelizumab for injection, Jiangsu Hengrui, China) plus anti-VEGF (apatinib for oral administration, Jiangsu Hengrui, China) at our institution were retrospectively reviewed. HCC was diagnosed pathologically or clinically based on Guidelines for Diagnosis and Treatment of Primary Liver Cancer in China [8]. The inclusion criteria for the study were as follows: Barcelona Clinic Liver Cancer (BCLC) Stage-C HCC, Child–Pugh score ≤7, and Eastern Cooperative Group (ECOG) performance status ≤1. The exclusion criteria were as follows: other specific systemic therapies for the primary cancer involved before receiving the combined treatment modality and patients lost to follow-up. Enrolled patients were classified as BCLC stage-C based on the presence of portal vein/hepatic vein invasion or extrahepatic metastasis, Child–Pugh class, and ECOG score. Ultimately, 38 patients were enrolled in this study. Treatment modality was determined according to multidisciplinary panel discussion, including hepatologists, hepatobiliary surgeons, radiologists, interventional radiologists, medical oncologists, and radiation oncologists.

Baselines clinical data were collected. These data included age, sex, etiology, Child–Pugh class, *α*-fetoprotein (AFP) level, ECOG performance status, vascular invasion and extrahepatic metastasis.

### 2.3. Intervention

TACE was initiated before camrelizumab or apatinib administration. The TACE procedure was performed via the femoral artery under local anesthesia. A 5-F catheter was introduced, and angiography was conducted to assess the tumor number, size, location, and tumor-feeding arteries. Then, chemotherapeutic agents at individualized doses based on the body mass index (lobaplatin, 30–50 mg; and epirubicin, 10–30 mg) were infused through the hepatic artery. Afterward, embolization via a microcatheter (2.7 F; Terumo Medical Corp., Tokyo, Japan; or 2.4 F; Merit Maestro, South Jordan, Utah, USA) either selectively or superselectively was performed with a conventional lipiodol-based technique. Post-TACE syndromes were recorded, and liver function indexes were checked within 1 week after each TACE session.

Camrelizumab and apatinib were administered initially 3–7 days after the elimination of post-TACE syndrome. Patients were administered 3 mg/kg camrelizumab intravenously, either every 3 weeks or every 4 weeks, and oral apatinib (250 mg per day). The discontinuation of or changes to the therapeutic regimen were considered based on disease progression, unacceptable adverse events (AEs), patient refusal, or clinician decision.

### 2.4. Outcomes

Tumor response, progression-free survival (PFS), overall survival (OS), and AEs were evaluated. Tumor response was assessed according to the modified response evaluation criteria for solid tumors (mRECIST), which included complete response, partial response, stable disease, and progressive disease [12]. ORR was defined as the proportion of patients who achieved a complete response and partial response and was evaluated at 2-3 months after initial therapy [12]. PFS was defined as the period from initial TACE treatment to disease progression or death. The OS time was defined as the period from initial TACE treatment to death or the end of the study period. AEs during the combination therapy were evaluated according to the Common Terminology Criteria for Adverse Events, version 5 [13].

### 2.5. Follow-Up

All patients were followed up regularly until the patient died or the end of the study period (July 31, 2021). Blood tests including complete blood count, liver function, renal function, thyroid function, and myocardial enzyme were performed every 3-4 weeks to monitor AEs related to camrelizumab and apatinib. If any adverse events occurred, the patients were asked to give feedback to the clinician or clinical team. During hospitalization, the adverse event profiles were reviewed and assessed. Tumor markers were assessed, and imaging examinations were performed every 1-2 months to evaluate the treatment response. When residual viable tumors were identified or new lesions developed, the patient was treated with TACE or medical care according to the tumor status and the patient's general condition.

### 2.6. Statistical Analysis

Continuous data are presented as the mean and standard deviation if normally distributed and median (range) and if the distribution is skewed for continuous variables. Categorical data are presented as frequencies and percentages. PFS and OS were estimated using the Kaplan–Meier method. Statistical analysis was performed using SPSS (Version 25.0, Chicago, IL) and GraphPad Software (Prism 8.0.1, San Diego, California).

## 3. Results

### 3.1. Patient Characteristics and Procedural Outcomes

The median age was 54.0 years, and 78.9% of patients were male. All patients were hepatitis B carriers. Twenty-six patients (68.4%) had an ECOG performance status of 0, and 12 patients (31.6%) had an ECOG performance status of 1. Thirty-five (92.1%) patients had Child–Pugh class A liver function, and three (7.9%) patients had Child–Pugh B7. The details of the patients' characteristics are given in Table 1.

Each patient received at least one TACE procedure, with a median of 3 sessions (range, 1–7). The median interval of TACE procedures was 4.0 months (range, 1.2–14.9). The median number of camrelizumab injections was 7 (range, 2–17). All patients received apatinib at the initial dose (250 mg per day), for a median duration of 5.2 months (range, 1.3–21.5 months). Three patients were administered a half dose (125 mg per day) due to intolerable apatinib-related AEs during the therapy.

### 3.2. Treatment Response and Overall Survival

**Figure 1:**
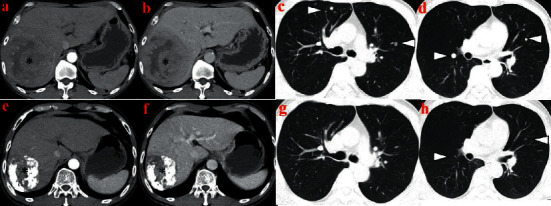
Representative imaging in advanced HCC patients receiving TACE combined with camrelizumab plus apatinib. (a-b) Enhanced abdominal CT showing massive HCC (asterisk). (c-d) Chest CT showing multiple lung metastases (white arrowhead). (e–h) At 3 months after combination therapy, follow-up CT demonstrated a partial response, with the tumor shrinking significantly in the liver (asterisk) and lung (white arrowhead).

In total, complete response was achieved in 1 patient (2.6%), partial response was achieved in 18 patients (47.4%), stable disease in 10 patients (26.3%), and progressive disease in 9 patients (23.7%). The ORR and DCR was 50.0% (19/38) and 76.3% (29/38), respectively. The tumor response is given in Table 2. One representative case of TACE combined with camrelizumab plus apatinib for the treatment of advanced HCC is shown in Figure 1.

**Figure 2:**
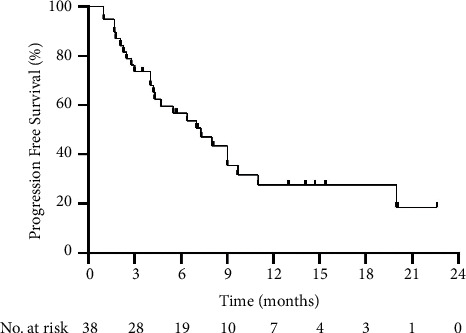
Kaplan–Meier curve of the progression-free survival time of all patients.

**Figure 3:**
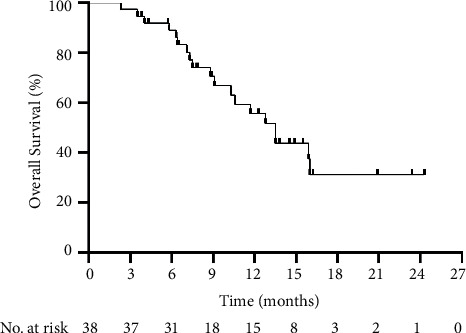
Kaplan–Meier curve of the overall survival time of all patients.

During a median follow-up of 10.6 months, 19 of the 38 patients (50.0%) died. The median PFS was 7.3 months (range: 1.0–22.6 months; Figure 2), and the median OS was 13.5 months (range: 2.3–24.3 months; Figure 3). The 6-, 9-, and 12-month OS rates were 81.6%, 47.4%, and 39.5%, respectively.

### 3.3. Adverse Events

Treatment-related AEs are given in Table 3. In total, 25 patients (67.8%) who received combination therapy had at least one treatment-related AE, including abdominal pain (*n* = 5), increased alanine aminotransferase/aspartate aminotransferase (*n* = 5), fever (*n* = 4), hypertension (*n* = 3), lymphopenia (*n* = 2), hand-foot skin reactions (*n* = 2), decreased appetite (*n* = 2), nausea/vomiting (*n* = 2), reactive cutaneous capillary endothelial proliferation (RCCEP, *n* = 1), diarrhea (*n* = 1), fatigue (*n* = 1), leukopenia (*n* = 1), neutropenia (*n* = 1), and anemia (*n* = 1). Notably, the most common AEs, such as abdominal pain, fever, and increased transaminase, predominantly were resulted from TACE. The camrelizumab/apatinib-related symptoms or signs related to the AEs in these patients were relieved or eliminated after symptomatic treatment, drug reduction, or interruption. No treatment-related deaths occurred.

## 4. Discussion

Multiple systemic agents have recently been approved for the treatment of unresectable HCC in both first- and second-line settings, increasing the therapeutic options for HCC patients [4, 6]. However, the efficacy of each monotherapy regimen is limited. In contrast, combination therapies show promise and demonstrate potential synergistic efficacy for advanced HCC. As new antitumor agents independently researched and developed in China, both apatinib and camrelizumab have shown effectiveness in the treatment of Chinese patients with unresectable HCC [7, 14, 15]. This retrospective study expands the treatment modality by the sequential application of camrelizumab plus apatinib after TACE for the treatment of advanced HCC and reports a remarkable ORR of 50.0% and DCR of 76.3%.

As is now well known, tumors grow and evolve through a constant crosstalk with the surrounding microenvironment, and emerging evidence indicates that angiogenesis and immunosuppression frequently occur simultaneously in response to this crosstalk [16]. Accordingly, strategies combining antiangiogenic agent and immunotherapy appear to have the potential to tip the balance of the tumor microenvironment and improve treatment response [16]. Based on our research and clinical practice, we believe that the encouraging results achieved here, by combining TACE treatment with antiangiogenesis and immunotherapy, are also due to the regulation effect of the tumor microenvironment after TACE procedure.

TACE is a feasible and effective treatment to enhance local tumor control in selected patients with advanced HCC, especially in Asian countries [8]. However, TACE can induce hypoxia and VEGF upregulation in HCC tissues, stimulating proangiogenesis and ultimately promoting tumor progression [17]. Herein, apatinib, as a small molecule TKI that selectively targets VEGF, could inhibit tumor neovascularization due to TACE and exert antitumor activity [15, 18, 19]. On the other hand, TACE itself could boost the changes in the profile of tumor-specific T cells by activating neotumor-associated antigens [20, 21]. Furthermore, anti-VEGF treatment was found to enhance anti-PD-1/anti-PD-L1 efficacy by reversing VEGF-mediated immunosuppression [22, 23]. As an ICI acting on the PD-1/PD-L1 pathway, camrelizumab combined with apatinib theoretically even promoted the synergistic antitumor effects in the TACE procedure.

In the current study, the median PFS and OS were 7.3 months and 13.5 months, respectively, which were better than those observed in monotherapy trials for advanced HCC patients [14, 18]. The preliminary results of several clinical trials evaluating the combination of ICIs and TKIs have recently been published. The phase 3 IMbrave150 trial revealed that atezolizumab and bevacizumab resulted in a better prognosis than sorafenib, with an ORR of 33.2% in unresectable HCC [5]. In the phase 2 RESCUE study, investigating the efficacy of apatinib plus camrelizumab in HCC patients, the ORR was 34.3% in the first-line setting [7]. Compared to these studies, the patients included in the present study had a relatively advanced stage. All patients were classified as BCLC stage-C, with a higher proportion of vascular invasion (71.1%) and extrahepatic metastasis (65.8%). Even so, the combined therapeutic regimen in our study yielded a relatively higher ORR of 50.0%, which confirmed the existence of the potential synergistic efficacy.

No unexpected AEs related to the combination therapy regimen were observed in this study. These AEs, such as hand-foot skin reaction, hypertension, and RCCEP, were predominantly related to apatinib or camrelizumab, while the other common AEs, such as abdominal pain, fever, and impaired liver function, were likely a result of TACE. Generally, camrelizumab possessed a safety profile and could be tolerated by the majority of patients [14]. In the current study, approximately 8% of patients discontinued apatinib treatment due to AEs. The incidence of apatinib-related grade ≥3 AEs in the current study was lower than that reported in previous studies [7, 18], which might have related to the relatively lower dose of apatinib used in our study. Overall, this combination regimen for patients with advanced HCC is safe and feasible in the clinic.

The current study also had some limitations. First, it was a retrospective study with a small sample of enrolled patients and a short follow-up period. Thus, the results may not be generalizable and should be interpreted with caution. However, the primary purpose of our study was to report the preliminary results of this triple therapy regimen. Given the advanced stage of patients in this study, this combination modality might exert maximal antitumor activity and could serve as first-line treatment option. Second, subsequent treatment after disease progression or unacceptable AEs was heterogeneous, which might have influenced the prognosis. In addition, the administration of camrelizumab on two different schedules could have contributed to the heterogeneity and potentially impacted patient outcomes. Despite these limitations, these results are encouraging and indicate the synergistic antitumor effect of TACE and camrelizumab plus apatinib in these patients.

In conclusion, the combination of TACE camrelizumab plus apatinib for the treatment of patients with advanced HCC showed encouraging efficacy with an acceptable safety profile. Further prospective studies are needed to confirm these results.

## Figures and Tables

**Table 1:** Patient characteristics (*n* = 38).

Variables	Median (range)/*n* (%)
Age (years)	54 (34–74)
Sex (male/female)	30 (78.9%)/8 (21.1%)
Etiology (HBV/others)	38 (100%)/0
ECOG score (0/1)	26 (68.4%)/12 (31.6%)
AFP (>400 ng/mL)	27 (71.1%)
Child–Pugh class (A/B)	35 (92.1%)/3 (7.9%)
Vascular invasion	27 (71.1%)
Extrahepatic metastasis	25 (65.8%)

HBV, hepatitis B virus; ECOG, Eastern Cooperative Oncology Group; AFP, *α*-fetoprotein.

**Table 2:** Response to combined therapy (*n* = 38).

Tumor response	*n* (%)
Complete response	1 (2.6)
Partial response	18 (47.4)
Stable disease	19 (26.3)
Progressive disease	9 (23.7)
Objective response rate	19/38 (50.0)
Disease control rate	29/38 (76.3)

**Table 3:** Adverse events (*n* = 38).

Adverse events	Grades 1-2	Grades 3-4	Any grade
All	38 (100%)	25 (67.8%)	38 (100%)
Hand-foot skin reactions	8 (21.1%)	2 (5.2%)	8 (26.3%)
Hypertension	10 (26.3%)	3 (7.9%)	13 (34.2%)
Diarrhea	7 (18.4%)	1 (2.6%)	8 (21.1%)
Rash	6 (15.8%)	0	6 (15.8%)
RCCEP	8 (21.1%)	1 (2.6%)	9 (23.7%)
Nausea and/or vomiting	30 (78.9%)	2 (5.2%)	32 (84.2%)
Fatigue	16 (42.1%)	1 (2.6%)	17 (44.7%)
Decreased appetite	33 (86.8%)	2 (5.2%)	35 (92.1%)
Fever	26 (68.4%)	4 (10.5%)	30 (78.9%)
Abdominal pain	27 (71.1%)	5 (13.2%)	32 (84.2%)
Increased ALT/AST	30 (78.9%)	5 (13.2%)	35 (92.1%)
Leukopenia	6 (15.8%)	1 (2.6%)	7 (18.4%)
Neutropenia	5 (13.2%)	1 (2.6%)	6 (15.8%)
Lymphopenia	4 (10.5%)	1 (2.6%)	5 (13.2%)
Thrombopenia	12 (31.6%)	0	12 (31.6%)
Anemia	7 (18.4%)	1 (2.6%)	8 (21.1%)

RCCEP, reactive cutaneous capillary endothelial proliferation; ALT, alanine aminotransferase; AST, aspartate aminotransferase.

## Data Availability

The data used to support the findings of this study are available from the corresponding author upon request.
